# The Role of Spirituality in Stroke Survivors and Their Caregivers: A Systematic Review

**DOI:** 10.1007/s10943-024-02029-0

**Published:** 2024-04-02

**Authors:** Rossella Ambrosca, Tatiana Bolgeo, Valentina Zeffiro, Rosaria Alvaro, Ercole Vellone, Gianluca Pucciarelli

**Affiliations:** 1 Department of Biomedicine and Prevention, University of Rome Tor Vergata, Via Montpellier 1, 00133 Rome, Italy; 2 Department of Activities Research Innovation, “San Antonio e Biagio e Cesare Arrigo” Hospital, Alessandria, Italy

**Keywords:** Spirituality, Stroke, Patient, Caregiver, Quality of life

## Abstract

Although several studies have analyzed the effects of spirituality on stroke survivors’ physical functioning and their caregiver’s outcomes, no systematic review or meta-synthesis was found to identify the lived experiences of stroke survivors and caregivers regarding spirituality. For these reasons, this study aimed to analyze quantitively and qualitatively research relating to stroke survivors’ and caregivers’ experiences. The included studies were RCT studies, quasi-experimental studies, qualitative, descriptive, ethnographic, and phenomenological studies, and cross-sectional studies (*n* = 37), with a total of 6850 stroke survivors and 1953 caregivers enrolled. Spirituality appears to play an important role in improving the quality of life and decreasing anxiety and depression of both stroke survivors and their caregivers.

**Supplementary Information:**

The online version contains supplementary material available at 10.1007/s10943-024-02029-0.

## Introduction

Stroke is a major cause of death that affects millions of people worldwide (Katan & Luft, 2018). The last report by the American Heart Association shows that someone dies of a stroke every 3 min and 33 s. Stroke is also a leading cause of severe long-term disability. In the USA, approximately 3% of males and 2% of females were disabled because of stroke (Virani et al., 2021). At the same time, in Europe, there are, on average, 1.12 million stroke episodes and 9.53 million individuals living with disabilities resulting from stroke episodes each year (Wafa et al., 2020a, 2020b). According to various studies (Feigin et al., 2009; Norrving & Kissela, 2013; Tsao et al., 2022), the number of cases is likely to increase more and more. In contrast, the number of deaths will decrease due to improved survival rates, leading to increased stroke survivors with psychological and physical problems related to the event (Farzadfar et al., 2011; Wafa et al., 2020a, 2020b).

Due to the increase in life expectancy, the number of people with stroke, and consequently, with post-stroke disabilities is increasing (King et al., 2020). Given the severe disabilities and limitations in activities of daily living that characterize the stroke, approximately 20% of stroke survivors experience anxiety (Knapp et al., 2017) and one-third of all stroke survivors suffer from depression either in the early or late period after stroke (Wu et al., 2019), associated with physical and psychological disabilities (Lenzi et al., 2008), social exclusion (Elloker & Rhoda, 2018), lower physical functioning (Vahlberg et al., 2017), poor rehabilitation outcomes (Baker et al., 2018) and increased mortality rate (Cai et al., 2019).

Furthermore, stroke affects not only the stroke survivors’ outcomes but indirectly also their caregivers’ outcomes. For example, as described in the literature, stroke caregivers experience many problems, such as anxiety, depression (Hu et al., 2018), exhaustion (Morais et al., 2012), hopelessness, fatigue (Morais et al., 2012), social isolation (Yu et al., 2013) and economic problems (Ugur & Erci, 2019). In addition, depression seems to be the most common symptomatology, which affects more than 40% of stroke caregivers (Loh et al., 2017), with a significant impact on both stroke survivors’ and caregivers’ quality of life (QOL) (Hu et al., 2018).

Although several authors tried to identify variables that could improve the stroke survivors’ and caregivers’ outcomes, such as anxiety (Rafsten et al., 2018), depression (Ezema et al., 2019), caregiver burden (Hu et al., 2018), QOL (Ramos-Lima et al., 2018) and physical functioning (Saunders et al., 2020), only a few authors have focused on analyzing the effect of spirituality in the stroke population (AbdAleati et al., 2016; Koenig, 2009; Moreira-Almeida et al., 2006). As described by the World Health Organization (WHO), spirituality is not a synonym of religiosity, but is described as a variable not related to the physical senses or external actions but to the higher faculties of the mind or higher moral qualities; and that it has a high refinement of thought or feeling (O'Connell & Skevington, 2010). Spirituality could be a powerful weapon against depression, increasing the levels of hope and trust in one’s potential and activating coping mechanisms (Ferreira-Valente et al., 2019) that are very useful in chronic diseases. In this context, the role of spirituality is increasingly developing in improving the QOL of individuals with chronic diseases (Burlacu et al., 2019; Reynolds et al., 2016) and their caregivers (Monteiro et al., 2018; Selman et al., 2018).

Despite stroke survivors and their caregivers having specific characteristics and no one chronic disease is the same as the others, from the data reported so far, studying the spirituality of stroke survivors and caregivers could be fundamental to improving holistically their care provided and increasing their perceived QOL. Pucciarelli et al. (2020), in their study, emphasize the importance of clinicians and nurses possessing knowledge on this topic to guarantee greater cultural competence in the health services and therefore greater support to the community, strengthening collaborative relationships between health organizations and faith-based organizations to the benefit of both survivors and their caregivers.

Although several systematic reviews (An & Shaughnessy, 2011; Cai et al., 2019; Tchero et al., 2018; Wray et al., 2018) were conducted on stroke survivors and their caregiver population, they do not focus on analyzing the role of spirituality in stroke survivor–caregiver dyads. Writing a systematic review on the topic of spirituality in the stroke survivor–caregiver dyads will ensure that healthcare professionals have the tools to educate stroke survivors and their caregivers. Comprehensive nursing care should also consider the spiritual well-being of stroke survivors and caregivers.

Therefore, the objectives of this systematic review are as follows:

1. Describe the effects of spirituality on the outcomes of stroke survivors and caregivers.
2. Conduct a meta-synthesis to identify the lived experiences of stroke survivors and caregivers regarding spirituality.

## Methods

### Design

A systematic literature review and a meta-synthesis were conducted by the Preferred Reporting Items for Systematic Reviews and Meta-Analyses (PRISMA) (Page et al., 2021) and Joanna Briggs Institute (JBI) guidelines (Vardell & Malloy, 2013). This systematic review was registered at PROSPERO (identification number: CRD42022382673). No approval from the Ethics Committee was needed due to the study's nature.

### Eligibility Criteria

#### Search Strategy

The PRISMA checklist was used to present detailed information in our systematic review. Each study was read and analyzed using the comparative tables of the Joanna Briggs Institute (JBI) (Vardell & Malloy, 2013).

A comprehensive search was conducted in June 2021. The databases analyzed to recruit the studies were: PubMed, Cinahl, Scopus, Web of Science, and PsycINFO, and the last consultation of the databases took place in November 2022. The keywords were as follows: “spirituality”, “religion”, “faith”, “stroke”, “patient”, “survivor”, “carer”, “dyad”, “caregiver”, “families”, and possible synonyms were also considered.

#### Criteria for Considering Studies for the Review

Studies eligible for the review had to fulfill some inclusion criteria. No time limit was set because, in the literature, no systematic reviews were found that dealt with the subject of this present study. The exclusion criteria were as follows: studies with topics other than specified above, in a language other than English or Italian, and not available in full text, focused on a pediatric population (if any), duplicate articles, studies not involving human participants (if any), studies in which participants’ diseases were generally identified as chronic. The title and abstract of studies identified by our search strategy were screened by two researchers (AR and TB) independently, and then they compared to see the results. The article review digital program Rayyan—Intelligent Systematic Review, was used to select articles by the blinded researchers. Discrepancies were resolved by discussion and a third reviewer (PG) undertook the role of supervisor. Concerning the checklist for randomized controlled trials (RCTs), thanks to the guidelines of the JBI we checked whether the case–control groups were equivalent, whether the participants were blindly assigned to the treatments, whether the analyses of the groups were identical, or if the reasons why this was not possible were specified.

About cross-sectional studies, using the JBI checklist we analyzed the reasons why researchers used a particular inclusion criterion, whether the study subjects and the setting were described clearly, how confounding factors were identified, and whether the statistical analysis was appropriate for the study.

In terms of the qualitative research studies, using the guidelines of the JBI checklist, we observed whether there was congruence between the stated philosophical perspective and the research methodology, on the latter and the methods for collecting the data, whether the representation of the results was consistent with the methodology and whether the voices of the participants were adequately represented.

All this information has been summarized in tables that will be attached to the study (Supplemental Tables I, II, and III) and can be consulted. The tables were drawn up individually by each researcher and in case of inconsistency, the supervisor played the role of mediator.

#### Population

The review included studies on the spirituality in stroke survivors, spirituality in caregivers of stroke survivors, and spirituality in stroke survivor–caregiver dyads.

#### Outcomes

The effects of spirituality on stroke survivors and caregivers were analyzed as medium-term (between three months and one year) outcomes. This choice was necessary because most of the included studies had medium-term outcomes. For stroke survivors, the analyzed outcomes were: QOL; activities of daily living; anxiety; depression; and stress. For caregivers, the analyzed outcomes were: QOL, stress; depression; and anxiety.

#### Study

The studies accepted to be included in the review were: RCT studies; quasi-experimental studies; qualitative analytical observational, descriptive, ethnographic, and phenomenological studies; and cross-sectional studies.

#### Assessment of the Methodological Quality and Risk of Bias

The selected studies were evaluated for methodological quality by two independent researchers using the critical appraisal tools made available by the Joanna Briggs Institute (JBI) to evaluate RCTs (JBI, 2023; Barker et al. 2023); this tool is shown in supplemental Table I.

The researchers used the Joanna Briggs Institute (JBI) Checklist for Qualitative Research to evaluate the qualitative studies (JBI, 2017a). This tool is shown in supplemental Table II.

Cross-sectional studies were analyzed using the Joanna Briggs Institute (JBI) Checklist for Analytical Cross-Sectional Studies/Observational Studies  (JBI, 2017a); this tool is shown in supplemental Table III.  Any disagreement between reviewers was resolved through discussion or consultation with a third reviewer. The JBI checklist for RCT studies made it possible to analyze the presence or absence of bias in the RCT studies selected; in particular, the correct randomization of the groups was analyzed along with whether the blind method was respected concerning the members belonging to the groups, the researchers and analysts of the results, along with an analysis of the proposed methodology used in the study and whether the correct statistical analysis of the results was obtained.

The JBI Checklist for qualitative studies analyzed the presence or absence of bias in studies included in this review, the congruity between the stated philosophical perspective and the research methodology, between the research methodology and the research question or objectives, between the research methodology and the representation and analysis of data, between the research methodology and the methods used to collect data, and the interpretation of the results. This tool was used to analyze the presence of researcher influence on the research and vice-versa, the presence of an adequate representation of participants’ voices, and the presence or absence of the research report flow.

The JBI Checklist for Analytical Cross-Sectional Studies analyzed whether: the inclusion criteria were clarified in the cross-sectional studies selected; the study subjects and the setting were described in detail; the data and results obtained were analyzed validly and reliably; the confounding factors were identified; and a correct statistical analysis was used.

The risk of bias in the included studies was assessed by two reviewers (AR and BT) using the JBI Critical Appraisal Checklist for RCT studies, the JBI Checklist for Qualitative Studies, and the JBI Checklist for Analytical Cross-Sectional Studies; supervision was by a third reviewer (PG). Based on these assessment tools, studies were rated as having a low, high, or unclear risk of bias.

#### Data Extraction

The data were extracted from the studies included in the review by two reviewers. The independently extracted data were: country; context of setting; characteristics of the participants; description of the group and sample; measured results; and description of the main outcome. The two different investigators (BT and AR) independently extracted the results based on the type of study, subsequently, the data were revised. If there were any differences they were resolved through debate, and if this failed, a third reviewer (PG) was consulted.

## Results

### Search Results

**Fig. 1:**
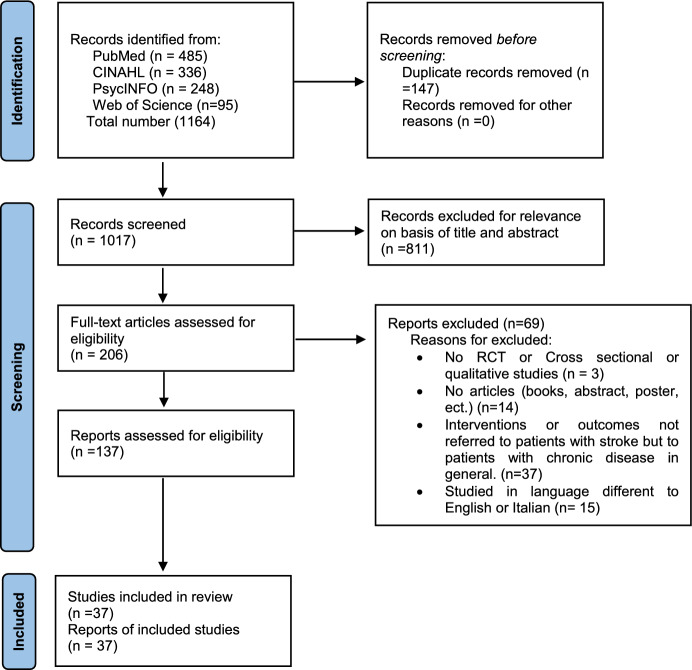
Flow Diagram of the systematic review

Literature searches identified 1164 records, after duplicates were removed (Fig. 1); 137 records were selected by titles because they fulfilled the inclusion criteria; after reading the full texts, 69 records were discarded. No study was excluded based on a quality critical appraisal tool. The included studies were published from 1995 to 2020.

### Methodology Quality and Risk of Bias in Included Studies

The methodological quality of qualitative, cross-sectional, and RCT studies is summarized in Table 1, 2, and 3. About qualitative studies, all of them obtained a score equal to 80 out of 100(Arnaert et al., 2006; Azar et al., 2022; Bays, 2001; Burns et al., 2022; Goetz, 2011; Goetz & Bloem, 2015; Jullamate et al., 2007; Laures-Gore et al., 2018; Materne et al., 2022; Moorley et al., 2016a, 2016b; Pierce, 2001; Pierce et al., 2008; Robinson-Smith, 2002; Strudwick & Morris, 2010). The main problem concerned the difficulty of understanding whether there had been a prejudice on the part of the researchers and therefore an influence of their ideas on the analysis of the results. Few studies summarized their results in tables but due to the nature of the study type, this is not a problem. Ten out of 16 studies had a score of 100 out of 100 (Arnaert et al., 2006; Azar et al., 2022; Bays, 2001; Burns et al., 2022; Laures-Gore et al., 2018; Materne et al., 2022; Pierce, 2001; Pierce et al., 2008; Robinson-Smith, 2002; Strudwick & Morris, 2010). In cross-sectional studies, the bias most found appears to be the identification of confounding factors, however, the majority of the studies clearly expressed the same. Specifically, 14 out of 18 studies obtained 100 out of 100 as a score (Alquwez & Alshahrani, 2021; Berges et al., 2007; Fauziah et al., 2022; Gholamzadeh et al., 2014; Giaquinto et al., 2007; Johnstone et al., 2008; Kes & Aydin Yildirim, 2020; Owolabi, 2011; Pucciarelli et al., 2020a, b; Rana et al., 2015; Safavi et al., 2019; Zauszniewski et al., 2021), following the evaluation of the checklist shown in Table 2. The last type of included studies were RCTs. The greatest risk with these types of studies was found to be the lack of sufficient descriptions of the randomization of the groups. About the blinding, not all the studies declared having been conducted in a double-blinded study and this could have generated errors in the analytical phase of the works. Of the six articles included, five scored above 80% (Fu et al., 2022; Ghous et al., 2017; Skolarus et al., 2012; Thrisna Dewi et al., 2020; Trihandini et al., 2018; Wong & Yeung, 2015), and one study scored 100% (Wong & Yeung, 2015). The lowest score was 76.9% (Trihandini et al., 2018) because the study was not conducted as a double-blinded study.

**Table 1:** Characteristics of qualitative studies

References (location)	Participants	Study design	Results
Bays (2001) (Kentuchy, USA)	9 stroke survivors (6 females and 3 males), mean age 68 years	Open-ended questions	*Hope*: Dynamic model based on goals and progress Means of connecting the stroke survivor to friends, family, other survivors
Pierce (2001) (Ohio, USA)	8 Primary caregivers and 16 secondary caregivers (4 males and 20 females), Age range 26–76	Semi-structured interview	*Caring allows the caregiver to* Be motivated Do a positive introspection Hope for the future Connect with the Surrounding environment Have a purpose
Robinson-Smith (2002) (North-east of USA)	8 stroke survivors (6 females, 2 males); age range: 57–85	Open-ended questions	*Prayer leads the stroke survivor to*: Connect with God and nature Focus on the present Improve rehabilitation Reduce the pain Lighten the burden of the disease
Arnaert et al. (2006) (Montreal, Canada)	8 stroke survivors (4 females and 4 males), age range: 19–90 years	Semi-structured interviews	*Hope allows the stroke survivor to* Improve the healing process Recognize the contributions of friends, family and health professionals
Jullamate et al. (2007) (Thailand, Asia)	20 stroke survivors (10 females, 10 males), age range: 60–90 years; 15 caregivers (15 females), age range: 28–79 years	Semi-structured Interviews	Among the reasons for caring for a stroke survivor, spirituality predominates *Spirituality leads the caregiver to*: Increase the empowerment of the stroke survivor and caregiver
Pierce et al. (2008) (Ohio, USA)	36 caregivers (gender not described), age range: 31–80 years	email chat	*Spirituality in caregivers of stroke patients, leads to*: Feel the presence a greater power Have the strength to go on Connect with nature Improve interaction with family members Feel grateful to those close to them during care
Studwick et al. (2010) (UK, Europe)	9 Informal caregivers (8 females, 1 male), mean age 62 years	Semi-structured interview	*The study highlights that spirituality in ethnic Caribbean caregivers leads to* Break down the social isolation perceived by this population Improve caring to stroke survivors
Goetz et al. (2011) (Michigan, USA)	15 stroke survivors (11 males, 4 females), age range: 49–79 years. 15 caregivers (gender and age range not described)	Open-ended questions	*Joining religious communities for caregivers and stroke survivors*: Reduces depression Reduces isolation Improve rehabilitation outcomes Improve coping strategies
Goetz et al. (2015) (Kentucky, USA)	12 stroke survivors (6 females, 6 males),( age range not described) and their caregivers (age range and gender not described)	Semi-structured interview	*The closeness of the religious community, in the dyad*: Improve coping in the early stages of home rehabilitation The burden of caregivers decreases
Moorley et al., (2016a, b) (UK, Europe)	7 stroke survivors (all females), age range: 47–82 years	Semi-structured interview	*Religion and belief in the stroke survivor bring*: Increased strength and determination to cope with the disease Consideration of God as a benevolent force that helps the survivor in sickness Recognition of the role of health professionals in the post stroke rehabilitation phase
Moorley et al., (2016a, b) (UK, Europe)	6 Stroke survivors (all females), age range: 47–87 years	Qualitative phenomenological design	*Spirituality for stroke survivors brings*: Improved acceptance of the pathology Evasion of the reasons put forward by medical personnel, which are difficult to accept
Rosyidah et al. (2018) (Indonesia, Asia)	7 stroke survivors (3 females, 4 males), age range: 34–68 years	Unstructured interview	*Spirituality brings*: Bond with God, through praying Trust in God, as a support for the disease Spiritual support in friends, relatives, family and health professionals Meaning and purpose Increased coping
Laures-Gore et al. (2018) (Georgia, USA)	13 Stroke survivors (7 females, 6 males) age range: 41–71 years	A structured interview	*Spirituality in the stroke survivor leads to*: Increased post stroke functional recovery Improved bonds with friends and family Increased feeling of goodwill toward those who support the survivor
Burns et al. (2022) (South-east of USA)	20 stroke survivors (15 females, 5 males), over 60 years age. 19 caregivers (14 females, 5 males), over 60 years age	A qualitative descriptive approach	*Faith for stroke survivors brings*: Healing
Matérne et al. (2022) (Sweden, Europe)	19 stroke survivors (10 male, 9 female), age range: 44–89 years and between 1 and 19 years post-stroke	Semi-structured interviews	*Life with stroke has been adapted to but not accepted. Five subthemes emerge*: Adapting and adjusting life, Meaningful values in life, Inner resources, Support and treatment from social relations, Support and treatment from external resources

**Table 2:** Characteristics of the cross-sectional studies included. Part 1

References (location)	Participants	Scales	Variables	Results
Berges et al. (2007) (Arizona, California, Colorado, New Mexico, and Texas; USA)	3050 noninstitutionalized Mexican- American stroke survivors	*Activities of daily living (ADLs)* *Instrumental activities of daily living (IADLs)* *Performance-Oriented Mobility Assessments (POMAs)* *Attendance at religious services*	*ADL* *IADL* *Mobility* *Spirituality*	+ ADLs and IADLs − Decline in lower body function
Giaquinto et al. (2007) (Italy, Europe)	132 stroke survivors (60 males, 72 females; mean age 70,0 years)	*Mini mental state examination* *Cumulative illness rating scale* *Functional independence measure scale* *Royal free interview (RFI)* *The Hospital Anxiety and Depression Scale (HADS)*	*HADS Spiritual and religious beliefs* MMSE	*Spirituality for stroke survivors leads to*: Protection from anxiety and disability-related depression
Johnstone et al. (2008) (Arizona, USA)	32 Stroke survivors	*Brief multidimensional measure of religiousness/spirituality* (*BMMRS*) *SF-36 health status questionnaire*	*Spirituality* *Quality of life*	Spiritual beliefs → potential protective factor against emotional distress
Qiu et al. (2008) (China, Asia)	A sample of 92 caregivers (66 females, 26 males)	*Short Portable Mental Status Questionnaire* (*SPMSQ*) *The five-item Barthel Index* (*BI*) *Brief COPE Inventory* (*BCI*) *The center for epidemiologic studies depression* (*CES-D*) *scale*	*Coping* *Depression* Functional Ability Mental status	*Religion for stroke survivors' caregivers such as*: Positive coping tool
Owolabi (2011) (Nigeria, Africa) (Germany, Europe)	40 females stroke survivors	*National Institute of Health Stroke Scale,* *Stroke Levity Scale (HRQOL)*	*Stroke severity* *Quality of life*	Spirituality → form of coping
Morgenstern et al. (2011) (Michigan, USA)	669 stroke survivors (48.7% were women, 37.8% of sample was between 60–74 years old)	*Mental Adjustment for Stroke Scale* *The Patient Health Questionnaire* *Strawbridge’s religiosity scale*	*Mental status* *Quality of life* *Spirituality*	Spirituality did not confer a significant effect on stroke severity, recurrence, or mortality
Gholamzadeh et al. (2014) (Iran, Asia)	96 caregivers of stroke survivors	*Brief Religious Coping Scale* (*Brief RCOPE*) *Index of Psychological Well-being*	Spirituality Well-being	*Spirituality for caregivers of stroke survivors brings*: Psychological well-being Better adaptation to the role of caregiver Reduces depression
Omu et al. (2014) (Kuwait, Asia)	40 females stroke survivors	*Santa Clara Strength of Religious Faith Questionnaire* (*SCSROF*) *The Psychosocial Adaptation Self-efficacy Scale* (PSE) *The Generalized Self-Efficacy Scale* (GSE) *Single item life satisfaction measure*	Religious faith, Self-efficacy, Life satisfaction	*Religious faith not significantly correlated with any of the main study variables*: GSE (Pearson r = 0.089), PSE (r = 0.114), and life satisfaction (r = 0.187)
Rana et al. (2015) (Germany, Europe) (Pakistan, Asia)	53 stroke survivors from Germany (average age 65 years, 69% males) and 44 from Pakistan(average age 62 years, 79% males)	*The Freiburg Questionnaire of Coping with illness* *The Short Form 12 Questionnaire (SF-12)* *The Survey of Social Support (F-SozU) of Fydrich *et al	Coping Religiosity Social support Quality of life	Religiosity and/or spirituality exert an influence on the type of coping as they promote active processing
Torabi Chafjiri et al. (2017) (Iran, Asia)	A sample of 407 caregivers of older stroke survivors in their family (362 females, mean age 38,8 years)	*The Spiritual Attitude Scale* *The Caregiver Burden Inventory*	Spirituality Caregiver Burden	+ Spirituality → − Caregiver burden
Safavi et al. (2019) (Iran, Asia)	120 Stroke survivors	Ross Religious Orientation scale World Health Organization Quality of Life Questionnaire (WHOQOL-BREF)	Spirituality Quality of life	+ Spirituality → +QOL
Ahrenfeldt et al. (2019) (10 countries of Europe)	927 stroke survivors (mean age 64,4 years)	Spirituality	Religious adherence socio-demographic data	*Spirituality as an affiliation to a religious organization brings*: Reduction in onset of heart attack, stroke (OR 0.68, 95% CI 0.50, 0.95) and diabetes
Alquwez et al. (2020) (Saudi Arabia, Asia)	A sample of 123 Informal caregivers of stroke survivors (68 males, 55 females)	*Spiritual Coping Strategies scale* *Multidimensional Scale of Perceived Social Support* *Hospital Anxiety and Depression Scale* (*HADS*) *The WHOQOL-BREF*	Social support Spirituality Quality of life HADS	*Religion for stroke survivors' caregivers leads to*: Good self-esteem Optimism
Kes et al. (2020) (Turkey, Asia)	181 Caregivers of stroke (mean age 45,90 years, 59% female)	*Religious coping scale* *Burden interview scale* *Family harmony scale–short form* (*FHS–SF5*)	Spirituality Burden Family Armony	*Religious coping of caregivers brings*: Increased family harmony Decrease in the burden of the caregiver Reduction of the care burden
Pucciarelli et al., (2020a, b) (Italy, Europe)	223 dyad stroke survivors and caregivers (Mean age of stroke survivors was 70.7; 104 females), (mean age of caregivers was 52.3, 140 females)	*WHOQOL- BRIEF (World Health Organization Quality of life-BRIEF)* *The Hospital Anxiety and Depression Scale* *WHOQOL-SRPB*	Spirituality Quality of life HADS	Spirituality moderated the effects of depression on stroke survivors’ and caregivers’ QOL
Pucciarelli et al., (2020a, b) (Italy, Europe)	414 stroke survivors (mean age 70.6 years; 52% males and 48% females)	*The WHOQOL-SRPB* *The WHOQOL-BRIEF*	Quality of life Spirituality	*The WHOQOL-SRPB scale is a more inclusive measure of spiritual dimensions because it analyzes personal beliefs, such as*: Awe, Integrity, integration, Hope, Optimism e Meaning of life
Zauszniewski et al. (2020) (Ohio, USA)	234 caregivers was enrolled in this study. (No information about age and gender)	*28-item Resourcefulness Scale* *23-item Delaney Spirituality Scale* *Burden Scale for Family Caregivers (BSFC-s)* *7-item and 8-item PROMIS scales*	Resourcefulness Spiritual practices Caregiver burden Anxious symptoms Depressive symptoms	The use of spiritual practices as coping resource has significant benefits on the caregiver and the care recipient’s health, including improved quality of life mental health
Fauziah et al. (2022) (Indonesia, Asia)	157 family caregivers (65.6% female)	*Barthel index scale* *Center for epidemiologic studies depression scale* *Revised scale for caregiving self-efficacy* *Relative's Questionnaire about Participation in Discharge Planning (R-QPD) scale* *Daily spiritual experience scale*	Physical functioning Depression Self-efficacy Spirituality	No significant association between caregiver depression and spiritual values was found in this study

**Table 3:** Characteristics of the RCTs

References (location) type of study	Participants	Interventions		Outcome measure	Results
Skolarus et al. (2012) (Texas, USA) RCT	710 stroke survivors	*The brain attack surveillance in corpus christi (BASIC)*: Active and passive surveillance were used to identify strokes In-person interview	*Usual care*: Ethnic differences in the pre-stroke prevalence of spirituality, optimism, depression and fatalism in a Mexican–American and non-Hispanic white stroke population	Outcomes at baseline *Patients*: Severity of stroke (NIHSS) Religiosity levels (Strawbridge's religiosity scale) Optimism's level (LOT-R) Depression (patient health questionnaire) Fatalism (Mental Adjustment for Stroke Scale (MASS) and Pearlin scale)	Greater spirituality and optimism are variables related to better recovery after stroke and more other survival rates in the intervention group
Wong et al. (2015) (China, Asia) RCT	108 stroke patients	*4-week transitional care programmed*: Home visits Telephone calls Holistic care interventions Transitional care track Holistic care managers	*Usual care*: Routine hospital-based physical training programmed during the first 3 weeks after hospital discharge	Outcomes at baseline and after 4 weeks. *Patients*: QOL (SF-36 scale) Functional performance (Modified barthel index) Depressive symptoms (CES-D) The patient satisfaction (PSQ-HK) Use of emergency room visits	The intervention group's spirituality support resulted in better spiritual-religious and personal measures *These data showed a correlation with*: Better quality of life Higher levels in the daily activities of stroke survivors
Ghous et al. (2017) (Pakistan, Asia) RCT	29 stroke patients (Mean age 54.4 years; 59% Males)	*Activity repetition training with Salat (prayer)*: The intervention was a tailored whole body training protocol providing 60 min/day, 4 days/week for 6 weeks with increasing number of repetitions	*Usual care*: Task-oriented training comprised numerous functional tasks designed to strengthen the upper extremity and certain activities for lower extremities in order to enhance walking balance, gait, speed and distance	Outcomes at baseline, after four and six weeks *Patients*: Motor function (motor assessment scale (MAS)) Balance (Berg Balance Scale) (BBS))	*The experimental group that carried out repetition-based exercise sessions with Salat had*: Clear improvement of the functional state, Mobility improvement e Increased balance
Trihandini et al. (2018) (Indonesia, Asia) Quasi-RCT	30 survivors of ischemic stroke	*Spiritual nursing care* Developed based on Swanson's theory of healing and O'Brien's dimensions of healing on the practice of spiritual healing. In three days, several strategies were adopted: Maintaining patient privacy, Greeting with eye contact and smiling, Introducing and sitting next to patients, Listening to the patient's experience, Focusing on the verbal and non-verbal expression of the patient's feelings, Answering their questions if necessary, Being with patients silently without speaking if patients were in pain and discomfort, Inform families to engage in care and motivate patients with reinforcing words, Clarify necessary things again before leaving the patient's room	*Usual care* (15 Patients)	Outcomes at baseline and after three days *Patients*: Anxiety (hamilton anxiety rating scale)	*Spiritual assistance in stroke survivors brings* Reduced anxiety post-test compared to pre-test *In the experimental group, the mean difference between pre- and post-test anxiety levels was 20.33 compared to 11.73 in the control group, demonstrating how spiritual assistance plays an important role in reducing anxiety levels.*
Thrisna Dewi et al. (2020) (Indonesia, Asia) Quasi-RCT	46 stroke survivors	The effects of Gayatri Mantra and Emotional Freedom Technique (EFT) on quality of life (QOL)	*Usual care*: Standard hospital rehabilitation program	Outcomes at baseline and after the intervention (after one day) *Patients*: QOL (SS-QOL)	A higher quality of life score was observed in the intervention group
Fu et al. (2022) (Zhengzhou, China)	68 stroke survivors and their caregivers	The benefit-finding intervention program consisted of 9 weeks of one-on-one interventions (per 45 min) with the participants, in their houses	*Usual care*: A routine health education	Zarit Caregiver Burden Interview Adult carer quality of life questionnaire Stroke-specific Quality of Life (SS-QOL)	The intervention appears to be feasible for stroke patients and caregivers. The intervention is capable of improving the quality of life of caregivers and survivors, increasing the benefit finding of caregivers and reducing the burden of caregivers

### Study Characteristics

The qualitative articles included in the study counted a total of 159 stroke survivors and 115 caregivers. The cross-sectional studies had 5744 stroke survivors and 1757 caregivers, and the RCT studies counted 991 stroke survivors and 68 caregivers. Sixteen studies were conducted in Asia: one study in Thailand (Jullamate et al., 2007); one in Turkey (Kes & Aydin Yildirim, 2020); two in Pakistan (Ghous et al., 2017; Rana et al., 2015); four in Iran (Gholamzadeh et al., 2014; Kes & Aydin Yildirim, 2020; Safavi et al., 2019; Torabi Chafjiri et al., 2017); one in Saudi Arabia (Alquwez & Alshahrani, 2021); three studies in China(Fu et al., 2022; Qiu & Li, 2008; Wong & Yeung, 2015); one in Kuwait(Omu et al., 2014); and three in Indonesia (Fauziah et al., 2022; Thrisna Dewi et al., 2020; Trihandini et al., 2018). Ten studies were conducted in Europe: three studies were carried out in Italy (Giaquinto et al., 2007; Pucciarelli et al., 2020a, b); three in England (Moorley et al., 2016a, b; Strudwick & Morris, 2010); one in Sweden (Materne et al., 2022); two in Germany(Owolabi, 2011; Rana et al., 2015); and one study was multicentre and was carried out in 10 European countries (Ahrenfeldt et al., 2019). Fourteen studies were conducted in the United States (Arnaert et al., 2006; Bays, 2001; Berges et al., 2007; Burns et al., 2022; Goetz, 2011; Goetz & Bloem, 2015; Johnstone et al., 2008; Laures-Gore et al., 2018; Morgenstern et al., 2011; Pierce, 2001; Pierce et al., 2008; Robinson-Smith, 2002; Skolarus et al., 2012; Zauszniewski et al., 2021), one study in Africa (Owolabi, 2011) and one in Canada (Arnaert et al., 2006).

#### Details of the Qualitative Studies’ Results

As for the qualitative studies, the tools used by the researchers were the use of questionnaires with open (Bays, 2001; Burns et al., 2022; Goetz, 2011; Robinson-Smith, 2002), semi-structured (Arnaert et al., 2006; Berges et al., 2007; Burns et al., 2022; Goetz & Bloem, 2015; Jullamate et al., 2007; Laures-Gore et al., 2018; Materne et al., 2022; C. R. Moorley et al., 2016a, b; Pierce, 2001; Strudwick & Morris, 2010) or structured(Rana et al., 2015) answers.

For all but one of the studies, interviews lasting an average of 60 min were conducted, in which researchers proposed questions and participants were free to speak or answer questions in writing. The study that did not use the interviews performed the qualitative analysis using a web email in which caregivers wrote and which was moderated by a specialist nurse (Pierce et al., 2008). Some studies have explicitly stated that they use data saturation (Arnaert et al., 2006; Azar et al., 2022; Bays, 2001; Jullamate et al., 2007; Materne et al., 2022; Pierce et al., 2008; Robinson-Smith, 2002) as a tool to terminate participant enrollment*.*

#### Results of Individual Studies

The results of the qualitative are reported in Table 1. Spirituality was seen as a means of connecting the stroke survivor to the present (Robinson-Smith, 2002), improving adaptive coping (Azar et al., 2022; Laures-Gore et al., 2018; Materne et al., 2022; Moorley et al., 2016a, b). As far as caregivers are concerned, spirituality has played a fundamental role in lasting care (Pierce, 2001; Strudwick & Morris, 2010) and in welcoming a loved one’s illness (Jullamate et al., 2007; Pierce et al., 2008). As for the caregiver–stroke survivor dyad, spirituality has had positive effects on both, reducing stress (Goetz, 2011), increasing acceptance of disease, reducing anxiety and depression, and improving the QOL of the dyad (Pucciarelli et al., 2020), and was also manifested through the closeness of the members of the religious community to which they belong (Goetz, 2011; Goetz & Bloem, 2015).

The results of the cross-sectional studies can be summarized in Table 2. Cross-sectional studies of stroke survivors have shown that spiritual coping has a positive influence on psychological function (psychological well-being and reduction of anxiety and depression) (Giaquinto et al., 2007; Johnstone et al., 2008; Pucciarelli et al., 2020), improving the stroke survivor’s adaptation to the post-stroke phase (Omu et al., 2014). Moreover, a study on the incidence of chronic diseases has shown that there is a positive relationship between spirituality and a decrease in the incidence of stroke (Ahrenfeldt et al., 2019). Cross-sectional studies conducted on caregivers have shown that positive spiritual coping improves the QOL of caregivers (Alquwez & Alshahrani, 2021; Zauszniewski et al., 2021), reduces anxiety and depression (Fauziah et al., 2022; Gholamzadeh et al., 2014), improves care (Torabi Chafjiri et al., 2017) and decreases perceived workload (Kes & Aydin Yildirim, 2020); conversely, negative coping strategies (e.g. seeing illness as a punishment from God, feeling plagued by the new condition) increase anxiety and depression (Gholamzadeh et al., 2014; Qiu & Li, 2008).

As described in Table 3, all subjects in the RCTs were stroke survivors. All included studies show that spirituality has positive effects concerning the variables analyzed (Ghous et al., 2017; Thrisna Dewi et al., 2020; Trihandini et al., 2018; Wong & Yeung, 2015). To summarize the included studies, we can divide the variables into two aspects: 1) *emotional–psychological*, which analyses QOL (Fu et al., 2022; Thrisna Dewi et al., 2020; Wong & Yeung, 2015) and the reduction of anxiety and depression (Owolabi, 2011; Skolarus et al., 2012; Trihandini et al., 2018; Wong & Yeung, 2015). Although the study interventions were different—such as nursing that complemented the stroke survivor’s spirituality (Trihandini et al., 2018), or the use of spiritual exercises (Thrisna Dewi et al., 2020; Wong & Yeung, 2015)—the results showed a reduction in anxiety and depression (Trihandini et al., 2018; Wong & Yeung, 2015) and an increase in QOL (Thrisna Dewi et al., 2020; Wong & Yeung, 2015). When the interventions of the studies were aimed at a comparison among multiple groups (Owolabi, 2011; Skolarus et al., 2012), the results showed that the sample with greater spirituality had a reduction in anxiety and depression (Owolabi, 2011; Skolarus et al., 2012) and an increase in QOL(Fu et al., 2022). 2) *Functional*, which analyzed balance and mobility (Ghous et al., 2017) and body functional standards (Wong & Yeung, 2015); in both these studies the results showed that by replacing standard rehabilitation exercises with spiritual techniques the variables studied improved.

#### Results of Syntheses

Analysis of the qualitative studies included in the review highlighted common themes.

##### Spirituality and Religiosity

Some studies observe the role that spirituality plays in stroke survivors. In some studies, spirituality is a positive factor: it is associated with an increase in coping strategies(Jullamate et al., 2007; Laures-Gore et al., 2018); it allows survivors to find meaning and purpose in illness; and it is seen as a synonym for health, in the sense of a perceived vital force in the life of the interviewee even after the stroke (Moorley et al., 2016). In one study (Moorley et al., 2016), it had a protective meaning: when people of Caribbean ethnicity did not accept the risk factors described by doctors, they attributed their illness to a spiritual factor, for example, a curse. Another study (Berges et al., 2007) showed that participants who participated in religious rites and enjoyed religious services were less likely to increase their disability.

##### Praying

The theme of praying has been predominant in some studies (Alquwez & Alshahrani, 2021; Azar et al., 2022; Pierce et al., 2008; Robinson-Smith, 2002). In a study (Robinson-Smith, 2002) for stroke survivors, it is seen as a tool of connection with God, family, friends, and nature. Praying is recognized by respondents as an integral part of rehabilitation. Praying allows them to feel more relief from the suffering of the disease.

#### Results of Quantitative Studies

About the cross-sectional studies, four were focused on stroke survivors (Ahrenfeldt et al., 2019; Giaquinto et al., 2007; Johnstone et al., 2008; Omu et al., 2014), five studies on caregivers(Alquwez & Alshahrani, 2021; Gholamzadeh et al., 2014; Kes & Aydin Yildirim, 2020; Qiu & Li, 2008; Torabi Chafjiri et al., 2017) and one study (Pucciarelli et al., 2020a, b) on stroke survivor–caregiver dyads.

##### Stroke Survivors

Studies (Ahrenfeldt et al., 2019; Giaquinto et al., 2007; Johnstone et al., 2008; Omu et al., 2014) that focused on stroke survivors, observed how spirituality could reduce stroke survivors’ stress and psychological function. Specifically, one study (Giaquinto et al., 2007) showed that prayer is a valid coping strategy for stroke survivors. Anxiety and depression increase with the severity of physical disability but in individuals who have a high spirituality, this does not happen. Spirituality leads to a reduction in emotional stress. Similarly, another study looked at the relationship between spiritual coping and psychological function, observing that spiritual coping serves as a protective factor against emotional distress following a stroke (Johnstone et al., 2008) Additionally, another study (Omu et al., 2014) observed that as stroke survivors age, spirituality increases. The latest multicentre study, conducted in Europe, enrolled multiple stroke survivors to analyze how spirituality influenced the pathology and found that participants’ affiliation with religious organizations was associated with a decrease in the incidence of stroke (Ahrenfeldt et al., 2019).

##### Caregivers

All cross-sectional studies (Alquwez & Alshahrani, 2021; Fauziah et al., 2022; Fu et al., 2022; Gholamzadeh et al., 2014; Kes & Aydin Yildirim, 2020; Pucciarelli et al., 2020; Qiu & Li, 2008; Torabi Chafjiri et al., 2017) enrolled showed that caregivers of stroke survivors performed their care work very intensively, for more than eight hours a day and often forgoing recreational activities. This intense activity of caring exposed caregivers to stress and depression, variables analyzed in each study enrolled. Regardless of the characteristics of the sample analyzed in the different studies, spirituality was a variable that influenced the life of the stroke survivor’s caregiver. In particular, in two studies (Gholamzadeh et al., 2014; Qiu & Li, 2008) it was found that caregivers can have two types of spirituality: a negative spiritual strategy (in which caregivers see stroke as a divine punishment, a plague that afflicts them), which leads to increased stress and anxiety levels, reducing their QOL; and a positive spiritual strategy (in which caregivers see stroke as part of God’s plan, a means of gaining strength) that has the opposite effects to the former. The researchers of the enrolled studies explained the positive impact of the positive spiritual coping strategy on caregivers’ QOL, which is that it guarantees an improvement in post-traumatic growth that has generated well-being in caregivers.

Studies also show that caregivers who applied positive religious coping strategies had a better relationship with stroke survivors, thus achieving a lower level of depression (Fauziah et al., 2022; Gholamzadeh et al., 2014). A study by Torabi Chafjiri et al. (2017), which analyzed the caregivers of stroke survivors, highlighted a negative relationship between spirituality and perceived workload. This for the authors is explained by spirituality not only influencing the mental and psychological state but also the physical one, increasing the ability to face crises. Spirituality for the caregivers enrolled in the study was the tool to cope with moments of stress and difficulties related to the care of stroke survivors, which led to a reduction in the levels of anxiety and depression in the caregivers themselves. These results were also highlighted in another study, conducted by Kes et al. (2020), in which the care burden of caregivers was negatively correlated with religious coping strategies(Kes & Aydin Yildirim, 2020). A final study, conducted by Alquwez et al. (2021), highlighted the importance of religious coping strategies in a population of Muslim caregivers of stroke survivors. Caregivers enrolled in the study used praying to accept the difficulties related to caring for their stroke-affected loved ones, which resulted in a reduction in depression and an increase in their QOL. This, according to the authors, was possible because prayer allowed caregivers to gain a sense of peace and acceptance of their condition (Alquwez & Alshahrani, 2021).

##### Stroke Survivor and Caregiver Dyads

The most recent included study showed that the use of the WHOQOL-SRPB scale is a more inclusive measure of the spiritual dimensions because it analyses personal beliefs, such as integration, hope, optimism and the meaning of life, as well as spirituality and religiosity, and which therefore can represent a valid tool for both the caregiver and the stroke survivor and for the interactions that one has on the other, in the analysis of these aspects (Pucciarelli et al., 2020a, 2020b).

All RCTs included in the review had stroke survivors as subjects except one (Fu et al., 2022). Themes common to several studies have emerged and will therefore be analyzed below by themes.

##### QOL, Anxiety, and Depression

The QOL of stroke survivors was a variable considered by two studies (Thrisna Dewi et al., 2020; Wong & Yeung, 2015), observing a correlation between improved QOL and interventions in the spiritual field. In particular, the study by Thrisna Dewi et al. (2020), showed that by replacing the standard rehabilitation strategies with prayer, in particular the recitation of the Gayatri Mantra, and by combining meditation with the use of the Emotional Freedom Technique, the subjects enrolled in the experimental study presented indicators of improvement in the QOL (Thrisna Dewi et al., 2020). This result, according to the authors, can be explained by the fact that stroke survivors are more motivated to exercise and benefit not only from the physical but also from the psychological sphere. The mean QOL index analyzed using the SS-QOL scale, specific for stroke survivors, increased in the intervention group from 148 to 153 in the postoperative period, while it remained unchanged in the pretest and the post-test of the control group (Thrisna Dewi et al., 2020). Wong et al. (2015), instead, observed in an RCT, that the experimental group had higher levels of spirituality than the control group. The questionnaire chosen by the researchers also looked at the spiritual and religious domains to calculate the QOL and the results showed that, although in the case group, that of stroke survivors, all domains were with a lower score than the control group, the domain of spirituality remained preserved, indeed in some individuals it was even increased compared to the control group. This showed, according to the authors, that stroke survivors enrolled in the study placed greater importance on the spiritual sphere than healthy individuals (Owolabi, 2011). The study by Trihandini et al. (2018), like the previous study, analyses the effects of the introduction of spirituality in nursing, choosing the anxiety of stroke survivors as the analyzed variable. While the control group received standard nursing care, the experimental group had meetings with a specialist nurse before discharge. During the meetings, the stroke survivor was allowed to express their spirituality. During the stage of setting up the rooms, a place was guaranteed where the stroke survivor could pray or meditate, interviews were held that allowed them to explore their spirituality. The results of the study demonstrated a significant reduction in anxiety levels. Specifically, the mean levels of anxiety (± SD) in the experimental group at the pretest were 29.33 ± 6.11, and at the post-test they were 9.00 ± 3.00, while for the control group, the difference was much less marked (mean pretest 29.47 ± 2.00 and post-test 17.73 ± 2.63) (Trihandini et al., 2018) The results of these RCT and quasi-RCT analyses demonstrated the important role of spirituality in nursing. For the health and well-being of the stroke survivor, if spiritual strategies are implemented, a better benefit is achieved for the stroke survivor.

##### Physical Function and Disability

One study analyzed the role of spirituality on stroke survivors’ physical recovery (Ghous et al., 2017; Owolabi, 2011). Specifically, the study conducted by Ghous et al. (2017), observed how the rehabilitation exercises proposed in the experimental group (exercises repeated for 60 min a day) based on the movements performed to recite the prayers (Salat), led to an increase in physical functioning, movement, and balance.

##### Reporting Biases

There may have been errors in the selection of articles, especially in the initial phase when the relevance of the articles occurred through the reading of the title and abstract. To minimize errors, the included studies were selected by two researchers and the inclusion of the articles was blinded. Another possible error may be secondary to the synthesis of the studies, that is to the choice of the predominant themes that dealt with the various articles. This may be true, especially for qualitative studies that developed several themes, to reduce the impact of this error, the various articles were divided by typology through the creation of tables that were elaborated by two distinct researchers and then shared. The only RCT that enrolled caregivers of stroke survivors demonstrated the utility of an intervention that included the spiritual aspect for caregivers. In the intervention group, there was a reduction in load levels and an increase in quality of life. The anxiety perceived by the caregivers was lower than in the control group and this increased the quality of life perceived by the enrolled subjects even more (Fu et al., 2022).

## Discussion

This study aimed to show the positive impact of spirituality on the life of the stroke survivors and their caregivers, and the positive effects of spirituality on the stroke survivor–caregiver dyads. In this systematic review, we observed that spirituality could have a positive effect on coping and on adherence to rehabilitation (Ghous et al., 2017; Owolabi, 2011), improving rehabilitation results, such as increased levels of balance and mobility (Ghous et al., 2017) and the functional state of the body (Wong & Yeung, 2015), increasing QOL (Owolabi, 2011; Safavi et al., 2019; Thrisna Dewi et al., 2020), reducing stroke survivors’ and caregivers’ anxiety and depression (Giaquinto et al., 2007; Johnstone et al., 2008; Skolarus et al., 2012; Wong & Yeung, 2015), and reducing the caregiving burden (Ahrenfeldt et al., 2019; Pierce et al., 2008; Strudwick & Morris, 2010).

As for stroke survivors, studies have shown the positive role of spirituality in adhering to treatment. Spirituality facilitates the acceptance of disabilities secondary to stroke. These positive effects from the studies analyzed are attributable to the fact that they see life after stroke as a second chance, as a gift that they must make the most of. Thus, stroke survivors with high spirituality achieve high motivation and focus, which brings higher standards of physical recovery and rigor to the therapeutic regimen.

Studies have also revealed the important role of spirituality in improving QOL and reducing anxiety and depression in stroke survivors. In a lot of included studies (Owolabi, 2011; Robinson-Smith, 2002; Safavi et al., 2019; Thrisna Dewi et al., 2020; Trihandini et al., 2018; Wong & Yeung, 2015), it was found that greater spirituality of stroke survivors led to better QOL. This phenomenon can be explained by the fact that the spirituality of stroke survivors is closely related to the increase in optimism, understood as a positive attitude toward the disease. This, from the analysis of the included articles, explains why stroke survivors who possessed a higher spirituality were focused not on their disease—that is, on what they could no longer do because of the stroke—but rather on having survived a potentially fatal disease. They saw their life as a new beginning, as a condition that humbled them by allowing them to enjoy the joys, they previously took for granted, such as being able to walk or eat independently. Pain was also seen as an element that allowed them to be close to God, an element that made them stronger. Conversely, people with low spirituality saw their life as a succession of pain and deprivation, feeling under constant punishment, thus increasing their anxiety about the future and, in turn, their depression.

About the spirituality in caregivers, from the articles included, it plays an essential role in reducing the burden of caregiving (Kes & Aydin Yildirim, 2020; Pierce, 2001; Strudwick & Morris, 2010; Torabi Chafjiri et al., 2017). This phenomenon is explained by the fact that, through spirituality, the focus of caregivers was not the free time they were deprived of or the fact they were having greater difficulties in the work or social sphere, which are the main elements that lead to burdening the caregivers of survivors of stroke, but rather taking care of loved ones as a leap of faith and better acknowledging the rehabilitation progress of stroke survivors in the post-stroke phase, recognizing that they have an active role in these advances. Caregivers who possessed higher levels of spirituality suffered less from anxiety and depression (Gholamzadeh et al., 2014), perceiving their lives as less stressful. This phenomenon is explained because, from what emerged in our study, caregivers with higher levels of spirituality have a better appreciation of the support provided by society and their families, being able to understand when it is necessary to ask for the support of health professionals. Furthermore, caregivers’ decision-making could be influenced by the relationship between the traditional role expectations of being a caregiver and religious/spiritual beliefs. Indeed, as observed by authors (Heath et al., 2018), although none of the enrolled caregivers specifically suggested that religious/spiritual factors were influential in their active caring, spiritual values may have been integrally influential during caregivers’ decision-making.

### Implications

This revision has several implications. Our study provides invaluable support to healthcare professionals involved in assisting stroke survivors and their caregivers to provide interventions that can implement their spirituality. Particularly in nursing, adopting a spiritual approach has proven to be of great help to stroke survivors in reducing anxiety and depression and improving their QOL (Trihandini et al., 2018; Wong & Yeung, 2015). Our study allows healthcare professionals to understand the risks to which stroke survivors who have low spirituality are subject, enabling them to act before their manifestation. The study will help healthcare professionals to act on developing caregivers’ spirituality to avoid burdens on those caring for stroke survivors and reduce the number of hospitalizations and relapses, which translates into lower care costs for survivors of stroke.

### Limitations

This study has several limitations. Firstly, although the selection of articles was conducted with methodological rigor, it is possible that some articles in the initial selection phase were excluded because their titles were misleading or not very explanatory. The second limitation is the number of caregivers, which is significantly lower than that of stroke survivors. Indeed, they represented only 11.92% of the total. The discrepancy between the two groups could be explained by the fact that in the literature there is greater attention to pathology, analyzing the role that spirituality plays in this, rather than on the well-being of the stroke survivor, for which it is essential to include the caregiver and, above all, the spiritual well-being of the caregiver. This substantial disparity between the number of caregivers and the number of stroke survivors enrolled is particularly true for RCT studies, in which the subjects analyzed were all survivors of a stroke. Going forward, it is desirable to conduct systematic reviews that analyze more studies with caregivers. The third limitation concerns the difficulty that has emerged in the discernment of studies that have dealt with spirituality with studies that have analyzed religiosity, often used interchangeably. It was therefore decided to include studies on religiosity and spirituality in order not to lose a large amount of information on the subject when it was clear in the articles that the reference to religiosity was a synonym for spirituality. In the end, the results, although interesting, remain sensitive to the size of the sample, the demographic characteristics of the clinical population, religious affiliation, and cultural context.

## Conclusions

Spirituality appears to have a positive association with reducing anxiety and depression in stroke survivors and their caregivers. Spirituality is also responsible for improving the QOL in both the groups analyzed and reducing the risk of mortality and recurrence of stroke in survivors. For further evidence and an ever-deeper exploration of the specific effects of spirituality on stroke survivors and their caregivers, future research is encouraged.

## Supplementary Information

Below is the link to the electronic supplementary material.

Supplementary file1 (DOCX 33 kb)
