# Supplementary material for: The Role of Spirituality in Stroke Survivors and Their Caregivers: A Systematic Review
Source: J Relig Health. 2024 Apr 2;63(5):3501–31. doi: 10.1007/s10943-024-02029-0 (PMC11502552; doi:10.1007/s10943-024-02029-0)
Supplement: Supplementary file 1 — Supplementary file1 (DOCX 33 kb) [file 10943_2024_2029_MOESM1_ESM.docx]

Supplemental Table I. Critical appraisal checklist for qualitative research

|  | Bays.1995 | Pierce. 2001 | Robinson-Smith .2002 | Arnaertet al.2006 | Jullamate et al. 2007 | Pierce et al.2008 | Studwick et al. 2010 | Goetz et al. 2011 | Goetz et al. 2015 | Moorley et al., 2016 | Moorley et al., 2016 | Rosyidah et al. 2018 | Laures-Gore et al. 2018 | Burns et al. 2019 | Azar et al. 2020 | Matérne et al., 2022 |
| --- | --- | --- | --- | --- | --- | --- | --- | --- | --- | --- | --- | --- | --- | --- | --- | --- |
| Is the congruity between the stated philosophical perspective and research methodology? | **+** | **+** | **+** | **+** | **+** | **+** | **+** | **?** | **+** | **+** | **+** | **+** | **+** | **+** | **+** | **+** |
| Is there congruity between the research methodology and the research question or objectives? | **+** | **+** | **+** | **+** | **+** | **+** | **+** | **+** | **+** | **+** | **+** | **+** | **+** | **+** | **+** | **+** |
| Is there congruity between the research methodology and the methods used to collect data? | **+** | **+** | **+** | **+** | **+** | **+** | **+** | **+** | **+** | **+** | **+** | **+** | **+** | **+** | **+** | **+** |
| Is there congruity between the research methodology and the representation and analysis of data? | **+** | **+** | **+** | **+** | **+** | **+** | **+** | **+** | **+** | **+** | **+** | **+** | **+** | **+** | **+** | **+** |
| Is there congruity between the research methodology and the interpretation of results? | **+** | **+** | **+** | **+** | **+** | **+** | **+** | **+** | **+** | **+** | **+** | **+** | **+** | **+** | **+** | **+** |
| Is there a statement locating the researcher culturally or theoretically? | **+** | **+** | **+** | **+** | **+** | **+** | **+** | **+** | **+** | **+** | **+** | **-** | **+** | **+** | **+** | **+** |
| Is the influence of the researcher on the research, and vice-versa, addressed? | **+** | **+** | **+** | **+** | **-** | **+** | **+** | **-** | **-** | **-** | **-** | **-** | **+** | **+** | **+** | **+** |
| Are participants, and their voices, adequately represented? | **+** | **+** | **+** | **+** | **+** | **+** | **+** | **+** | **+** | **+** | **+** | **+** | **+** | **+** | **+** | **+** |
| Is the research ethical according to current criteria or, for recent studies, and is there evidence of ethical approval by an appropriate body? | **+** | **+** | **+** | **+** | **+** | **+** | **+** | **+** | **+** | **+** | **+** | **+** | **+** | **+** | **+** | **+** |
| Do the conclusions drawn in the research report flow from the analysis, or interpretation, of the data? | **+** | **+** | **+** | **+** | **-** | **+** | **+** | **+** | **+** | **+** | **+** | **+** | **+** | **+** | **+** | **+** |
| **Total** | **100** | **100** | **100** | **100** | **80** | **100** | **100** | **80** | **90** | **90** | **90** | **90** | **100** | **100** | **100** | **100** |

Note. + = yes; - = not; ? = Unclear/Not applicable

Supplemental Table II. Critical Appraisal for observational studies

|  | Berges et al. 2007 | Giaquinto et al. 2007 | Johnstone et al. 2008 | Qiu et al. 2008 | Owolabi, 2011 | Morgenstern et al., 2011 | Gholamzadeh et al. 2014 | Omu et al. 2014 | Rana et el. 2015 | Torabi Chafjiri et al. 2017 | Safavi et al. 2019 | Ahrenfeldt et al. 2019 | Alquwez et al. 2020 | Kes et al. 2020 | Pucciarelli et al. 2020 | Pucciarelli et al. 2020 | Zauszniewski et al., 2020 | Fauziah et al., 2022 |
| --- | --- | --- | --- | --- | --- | --- | --- | --- | --- | --- | --- | --- | --- | --- | --- | --- | --- | --- |
| Were the criteria for inclusion in the sample clearly defined? | + | + | + | + | + | + | + | + | + | + | + | + | + | + | + | + | + | + |
| Were the study subjects and the setting described in detail? | + | + | + | + | + | + | + | + | + | + | + | + | + | + | + | + | + | + |
| Was the exposure measured in a valid and reliable way? | + | + | + | + | + | + | + | + | + | + | + | + | + | + | + | + | + | + |
| Were objective, standard criteria used for measurement of the condition? | + | + | + | + | + | + | + | + | + | + | + | + | + | + | + | + | + | + |
| Were confounding factors identified? | + | + | + | ? | + | + | + | - | + | ? | + | + | + | + | + | + | + | + |
| Were strategies to deal with confounding factors stated? | + | + | + | - | + | ? | + | - | + | ? | + | + | + | + | + | + | + | + |
| Were the outcomes measured in a valid and reliable way? | + | + | + | + | + | + | + | + | + | + | + | + | + | + | + | + | + | + |
| Was appropriate statistical analysis used? | + | + | + | + | + | + | + | + | + | + | + | + | + | + | + | + | + | + |
| **Total** | **100** | **100** | **100** | **75** | **100** | **87,5** | **100** | **75** | **100** | **75** | **100** | **100** | **100** | **100** | **100** | **100** | **100** | **100** |

Note. + = yes; - = not; ? = Unclear/Not applicable

Supplemental Table III. Critical appraisal for RCT

|  | Skolarus et al. 2012 | Wong et al. 2015 | Ghous et al. 2017 | Trihandini et al. 2018 | Thrisna Dewi et al. 2020 | Fu et al. 2020 |
| --- | --- | --- | --- | --- | --- | --- |
| Was true randomization used for assignment of participants to treatment groups? | + | + | + | ? | + | + |
| Was allocation to treatment groups concealed? | + | + | + | + | + | + |
| Were treatment groups similar at the baseline? | + | + | + | + | + | + |
| Were participants blind to treatment assignment? | + | + | + | + | + | + |
| Were those delivering treatment blind to treatment assignment? | + | + | + | ? | + | - |
| Were outcomes assessors blind to treatment assignment? | ? | + | ? | ? | ? | - |
| Were treatment groups treated identically other than the intervention of interest? | + | + | + | + | + | + |
| Was follow up complete and if not, were differences between groups in terms of their follow up adequately described and analyzed? | + | + | + | + | + | + |
| Were participants analyzed in the groups to which they were randomized? | + | + | + | + | + | + |
| Were outcomes measured in the same way for treatment groups? | + | + | + | + | + | + |
| Were outcomes measured in a reliable way? | + | + | + | + | + | + |
| Was appropriate statistical analysis used? | + | + | + | + | + | + |
| Was the trial design appropriate, and any deviations from the standard RCT design (individual randomization, parallel groups) accounted for in the conduct and analysis of the trial? | + | + | + | + | + | + |
| **Total** | **92.3** | **100** | **92.3** | **76.9** | **92.3** | **84,6** |

Note. + = yes; - = not; ? = Unclear/Not applicable
